# TXNIP induces growth arrest and enhances ABT263‐induced apoptosis in mixed‐lineage leukemia‐rearranged acute myeloid leukemia cells

**DOI:** 10.1002/2211-5463.12908

**Published:** 2020-06-24

**Authors:** Mina Noura, Hidemasa Matsuo, Asami Koyama, Souichi Adachi, Hiroshi Masutani

**Affiliations:** ^1^ Department of Human Health Sciences Graduate School of Medicine Kyoto University Kyoto Japan; ^2^ Department of Clinical Laboratory Sciences Tenri Health Care University Tenri Japan; ^3^ Department of Pediatrics Graduate School of Medicine Kyoto University Kyoto Japan

**Keywords:** AML, autophagy, MLL, TXNIP

## Abstract

Thioredoxin‐interacting protein (TXNIP) has been widely recognized as a tumor suppressor in various cancers, including liver, breast, and thyroid cancers. Although TXNIP is epigenetically silenced in acute myeloid leukemia (AML) cells, as in many cancer cells, its role in leukemogenesis remains elusive. Mixed‐lineage leukemia (MLL) gene rearrangements in AML are associated with poor prognosis, and the development of a new treatment method is eagerly anticipated. In this study, we first reveal that lower expression of TXNIP is correlated with shortened overall survival periods in AML patients. Moreover, we demonstrated that TXNIP overexpression significantly suppresses proliferation in AML cells harboring MLL fusion genes. TXNIP promotes autophagy by increasing expression of the autophagy protein, Beclin 1, and lipidation of LC3B. We also show that TXNIP overexpression combined with ABT263, a potent inhibitor of Bcl‐2 and Bcl‐xL, is highly effective at inducing cell death in MLL‐rearranged (MLL‐r) AML cells. In summary, this study provides insights into the molecular mechanism of TXNIP‐mediated tumor suppression and furthermore underscores the potential of TXNIP as a promising therapeutic target for MLL‐r AML.

## Graphical abstract

Thioredoxin interacting protein (TXNIP) has been well known as a tumor suppressor in various cancers, including acute myeloid leukemia (AML). In this study, we found TXNIP overexpression causes growth arrest in mixed‐lineage leukemia‐rearranged AML cells. TXNIP induces autophagy by increasing expression of Beclin 1. Moreover, TXNIP overexpression‐induced autophagy increases sensitivity to the Bcl‐2 and Bcl‐xL inhibitor, ABT263, by promoting apoptosis induction.

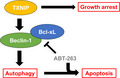

**Abbreviations**
AML: acute myeloid leukemia
MLL: mixed‐lineage leukemia
TXNIP: thioredoxin‐interacting protein
VDUP‐1: vitamin D3 upregulated protein 1

Acute myeloid leukemia (AML) is a malignant disease, in which immature blood cells aberrantly expand in the bone marrow and lose their normal hematopoietic function. Although the treatment outcome of AML is improving, the 5‐year recurrence‐free survival rate of adult AML is about 40% [1]. In addition, AML therapeutic sensitivity and prognosis are greatly different depending on the subtypes of leukemia and the underlying genetic abnormality. The standard treatment for AML is chemotherapy with a combination of cytarabine and anthracycline; however, it often results in poor prognosis due to the acquired resistance [2]. Mixed‐lineage leukemia (MLL)‐rearranged (MLL‐r) leukemia is characterized by chromosomal translocations of MLL gene, located at 11q23, to form fusion proteins with various partner proteins [3]. Rearrangements of MLL gene are found in 5–10% of all acute leukemia and in up to 85% of secondary leukemia after treatment with DNA topoisomerase II inhibitors, often accompanied with unfavorable prognosis [2, 4]. Therefore, it is eagerly desired to identify the underlying factors of malignancy and to develop a new therapeutic strategy for refractory AML, such as MLL‐r AML.

We identified thioredoxin‐binding protein 2, also called thioredoxin‐interacting protein (TXNIP) or vitamin D3 upregulated protein 1 (VDUP‐1), as a negative regulator of the key antioxidant system thioredoxin (TRX) [5]. TXNIP plays a role in a wide variety of physiological processes [6] including glucose metabolism [7, 8, 9], lipid metabolism [10, 11], and immune and inflammatory responses [12, 13, 14]. We previously reported that TXNIP knockout mice were predisposed to death under fasting conditions due to hyperlipidemia, hypoglycemia, bleeding tendency, and hepato‐renal insufficiency [11]. These results show that TXNIP is essential in the fasting response. Furthermore, TXNIP plays important roles in regulating cell death, growth, and differentiations. A number of studies have shown that TXNIP expression is decreased in various cancer cells. Loss of TXNIP in mice promoted hepatocarcinogenesis [15]. Disruption of TXNIP is important for the development of breast and thyroid cancers [16, 17]. Downregulation of TXNIP promotes tumor proliferation, migration, and metastasis by remodeling of extracellular matrix [18]. In adult HTLV‐1 T‐cell leukemia (ATL), the loss of TXNIP expression is involved in T‐cell leukemia progression [19]. Decreased expression of TXNIP has also been reported in AML cells. TXNIP is silenced in AML cells by two epigenetic regulators, histone deacetylation and H3K27me [20]. These studies suggest that TXNIP is involved in AML development, but the precise mechanism is unknown.

Autophagy is a self‐digesting mechanism, in which cytoplasmic components including organelles are sequestered within double‐layer membrane that delivers their contents to lysosomes for degradation [21, 22]. Although autophagy is conspicuously activated through starvation, it is induced at low levels under eutrophic conditions to maintain cellular homeostasis [23, 24]. The role of autophagy in cancer is complicated because of its dual functions as a tumor suppressor and promoter. Deficiency of autophagy in mice causes multiple liver tumors [25, 26]. However, autophagy accelerates the tumorigenesis of K‐Ras^G12D^‐driven lung cancer [27, 28]. These studies suggest that whether autophagy works for cancer suppression or promotion depends on the type of tissue, genetic background, and the stage of tumor development. TXNIP is reported to promote autophagy by interacting with autophagy regulator REDD1 [29, 30], suggesting that TXNIP may trigger autophagic death in AML cells. In spite of these findings, the precise molecular mechanisms behind TXNIP tumor suppressor activity and the functional association between TXNIP and autophagy have remained entirely elusive.

In this study, we compared the prognosis of AML subtypes between TXNIP low and high groups and the expression levels of TXNIP. Besides, we examined the effect of TXNIP overexpression on the cell growth of MLL‐r AML cells. These findings propose a promising strategy for AML with poor prognosis such as MLL‐r AML.

## Materials and methods

### Cell lines

MLL‐r AML‐derived MOLM‐13 and MV4‐11 cells were purchased from Deutsche Sammlung von Mikroorganismen und Zellkulturen GmbH (DSMZ, Braunschweig, Germany), and American Type Culture Collection (ATCC, Manassas, VA, USA), respectively. These MLL‐r AML cell lines were cultured in Roswell Park Memorial Institute 1640 medium containing 10% heat‐inactivated FBS and 1% penicillin–streptomycin (PS) under 5% CO_2_ and 95% air at 37 °C. Embryonic kidney HEK293T cells were obtained from Japanese Collection of Research Bioresources (JCRB, Ibaraki, Osaka, Japan). HEK293T cells were maintained in Dulbecco's modified Eagle's medium supplemented with 10% FBS and 1% PS under 5% CO_2_ and 95% air at 37 °C.

### Expression plasmids

Human TXNIP cDNA was amplified by PCR and then inserted into CSIV‐TRE‐Ubc‐KT expression vector (RIKEN, BRC, Tsukuba, Ibaraki, Japan). All the PCR products were verified through DNA sequencing.

### Production and transduction of lentivirus

For the production of lentivirus, HEK293T cells were transiently cotransfected with packaging and envelope plasmids, such as psPAX2 and pMD2.G, by polyethylenimine (Sigma‐Aldrich, St. Louis, MO, USA). Forty‐eight hours after transfection, viral supernatants were collected and immediately used for infection, and the successfully transduced cells were then sorted using flow cytometer Aria III (BD Biosciences, Franklin Lakes, NJ, USA) based on immunofluorescence (Kusabira‐Orange).

### Cell proliferation

To assess cell proliferation, 5 × 10^4^ cells of MOLM‐13 and MV4‐11 cells were seeded in 6‐well plates. For tetracycline‐inducible gene, doxycycline was added to the culture at a final concentration of 3 µm. Trypan blue dye exclusion assays were performed every other day.

### Immunoblotting

Cells were washed in PBS and then lysed in Triton X‐100 lysis buffer (20 mm Tris/HCl pH 7.5, 150 mm NaCl, 1% Triton X‐100, protease inhibitor EDTA free). After centrifugation, the protein content of the supernatants was measured using DC Protein Assay (Bio‐Rad Laboratories). Equal amounts of whole‐cell lysates were separated by SDS/PAGE and then electrotransferred onto polyvinylidene difluoride membranes. Membranes were probed with the following primary antibodies: anti‐VDUP‐1 (Invitrogen, 40‐3700, Carisbad, CA, USA), anti‐GAPDH (Santa Cruz Biotechnology, Inc., 0411, Dallas, TX, USA), anti‐LC3B (Cell Signaling Technology, #3738, Danvers, Essex, MA, USA), and anti‐BECN1 (Cell Signaling Technology, #2775). For secondary antibodies, HRP‐conjugated anti‐rabbit IgG and anti‐mouse IgG (GE Healthcare Life Sciences, Marlborough, MA, USA) were used. Blots were visualized using Chemi‐Lumi One Super (Nacalai Tesque) and ChemiDoc XRS + Imager (Bio‐Rad Laboratories, Hercules, CA, USA) according to the manufacturer's recommendations.

### Transmission Electron Microscope (TEM)

Acute myeloid leukemia cells were collected by centrifugation, fixed in 0.1 m phosphate buffer containing 4% paraformaldehyde/2% glutaraldehyde at 4 °C, washed in isotonic phosphate‐buffered sucrose, and then refixed in phosphate‐buffered 1% OsO_4_. Following dehydration in a graded series of EtOH washes, the cells were embedded in LUVEAK‐812 (Nacalai Tesque). Thin sections (70–90 nm thick) were cut on EM UC6 Ultramicrotome using a diamond knife (Leica, Heidelberg, Germany), stained with uranyl acetate and lead citrate, and then observed under H‐7650 electron microscope (Hitachi, Tokyo, Japan).

### Reagents

ABT263 was purchased from Cayman Chemical (Ann Arbor, MI, USA).

### Cell cycle and apoptosis assay

For cell cycle analysis, cells were fixed in 100% EtOH followed by incubation with PBS containing 100 µg·mL^−1^ of RNase A. Cells were then stained with 50 µg·mL^−1^ propidium iodide and subjected to flow cytometric analysis. For apoptosis assay, apoptotic cells were detected by annexin V–FITC conjugate (Invitrogen). In brief, ~ 2 × 10^5^ cells were washed in PBS, suspended in annexin‐binding buffer, and then mixed with 5 µL of annexin V. The reaction mixtures were incubated for 30 min. After incubation, cells were diluted, stained with 5 µg·mL^−1^ of DAPI (Invitrogen) or 5 µg·mL^−1^ of Hoechst 33342 (Invitrogen), and processed for flow cytometric analysis.

### IC50 evaluation

For cell survival assay, cells were seeded at a density of 1 × 10^5^ cells per mL. ABT263 was added to the culture medium, and cells were incubated for 48 h. Cell viability was then assessed using Cell Count Reagent CellTiter 96 AQueous One Solution Cell Proliferation Assay System (Promega, Madison, WI, USA) and VersaMax microplate reader (Molecular Devices, San Jose, CA, USA) according to manufacturer's instructions. Percent inhibition curves were drawn, and IC50 of ABT263 was calculated based on the median‐effect method [31].

### Statistics

Statistically significant differences between control and experimental groups were assessed using 2‐tailed unpaired Student’s *t‐*test and showed if the *P* value was < 0.05. Equality of variances in two populations was calculated with *F*‐test. The results were represented as the average ± SEM values obtained from three independent experiments.

PrognoScan software (http://gibk21.bse.kyutech.ac.jp/PrognoScan/index.html) was utilized for data extraction and calculation of minimal *P* value [32]. Survival between the indicated groups was compared using log‐rank test.

## Results

### TXNIP overexpression exerts antileukemic effect

**Fig. 1:**
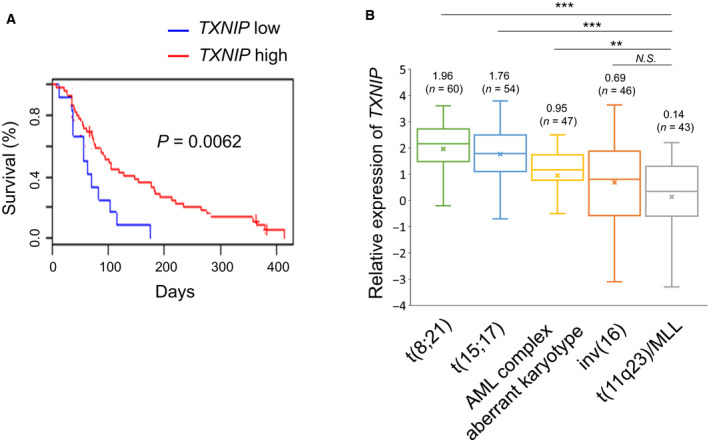
Lower expression level of TXNIP is associated with poor prognosis. (A) Overall survival of relapsed and refractory AML patients with higher or lower expression levels of *TXNIP* (GSE5122, high *n* = 46, low *n* = 12). *P* value by log‐rank (Mantel–Cox) test. (B) Expression levels of *TXNIP* in indicated primary AML cells (GSE6891). AML cells are compared to their nearest normal counterpart. Data are expressed as mean ± SEM values. **P < 0.01, ***P < 0.001, N.S., not significant, by 2‐tailed Student’s *t‐*test.

**Fig. 2:**
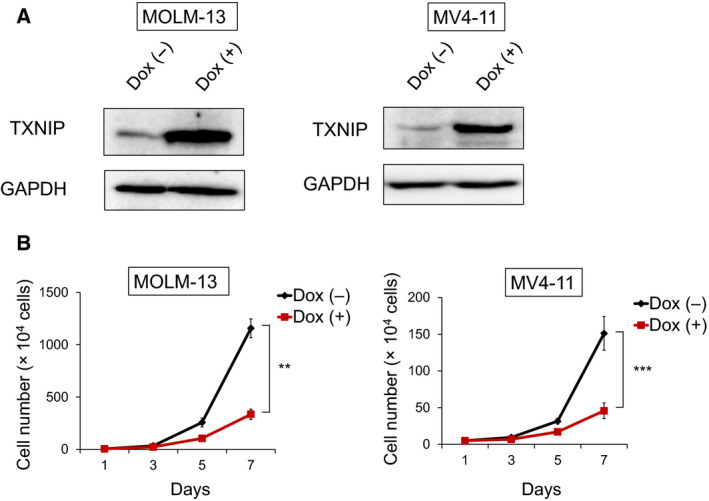
Doxycycline‐induced TXNIP suppresses proliferation of MLL‐r AML cells. (A) Expression of TXNIP in doxycycline (dox)‐on‐dependent TXNIP‐overexpressed MOLM‐13 and MV4‐11 cells. Cells were treated with or without 3 μm doxycycline. Seventy‐two hours after treatment, cell lysates were prepared and analyzed by immunoblotting with the indicated antibodies. GAPDH was used as loading control. Images are representative images of three reproducible independent results. (B) Growth curves of dox‐on‐dependent TXNIP‐overexpressed MOLM‐13 and MV4‐11 cells treated with or without doxycycline (*n* = 3). Data are expressed as the mean ± SEM values. **P < 0.01, ***P < 0.001, by 2‐tailed Student’s *t‐*test.

We first analyzed the clinical datasets, and we revealed that lower TXNIP expression was associated with poor prognosis in patients with refractory AML and relapsed AML (Fig. 1A). To investigate whether TXNIP expression is different among AML subgroups, we further analyzed clinical datasets and found that TXNIP expression was most decreased in MLL‐r AML cells (Fig. 1B). We thus decided to perform functional analysis of TXNIP using MLL‐fused AML cell lines, MOLM‐13 and MV4‐11. Both cell lines were obtained from MLL‐r AML patients with high‐risk factor FLT‐3 ITD. To investigate TXNIP overexpression‐mediated cellular responses, we constructed tetracycline‐inducible TXNIP expression vector and lentivirally transduced it into MOLM‐13 and MV4‐11 (Fig. 2A). As shown in Fig. 2B, TXNIP overexpression significantly suppressed MOLM‐13 and MV4‐11 cell proliferation. These data indicate that decreased expression of TXNIP confers leukemogenesis.

**Fig. 3:**
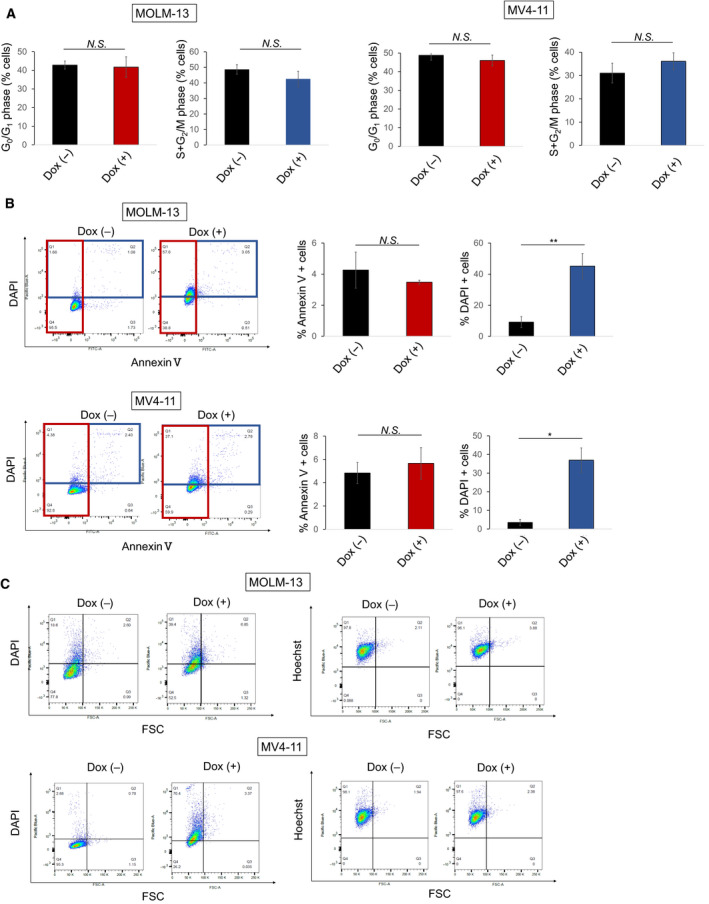
TXNIP regulates plasma membrane permeabilization. (A) TXNIP overexpression does not change the distribution of cell cycle stages. Dox‐on‐dependent TXNIP‐overexpressed MOLM‐13 and MV4‐11 cells were treated with or without 3 μm doxycycline. Cells were harvested and analyzed by flow cytometry 72 h after treatment (*n* = 3). (B) TXNIP overexpression does not affect the number of annexin V‐positive cells but affects the number of DAPI‐positive cells. Dox‐on‐dependent TXNIP‐overexpressed MOLM‐13 and MV4‐11 cells were treated as in A, and the number of cells stained with indicated fluorescent probes was scored by flow cytometric analysis (*n* = 3). (C) TXNIP overexpression does not influence the number of Hoechst 33342‐positive cells. Dox‐on‐dependent TXNIP‐overexpressed MOLM‐13 and MV4‐11 cells were treated as in A, and the number of cells stained with indicated fluorescent probes was scored by flow cytometric analysis (*n* = 1). Data are expressed as mean ± SEM values. *P < 0.05, **P < 0.01, N.S., not significant, by 2‐tailed Student’s *t‐*test.

To investigate the mechanism of growth suppression by TXNIP, we first performed cell cycle analysis and apoptosis assay. TXNIP overexpression changed neither the distribution of cell cycle stages nor the number of apoptotic cells (Fig. 3A,B). Meanwhile, TXNIP overexpression notably increased the number of annexin V‐negative/DAPI‐positive cells (Fig. 3B). These data suggest that TXNIP enhanced the permeability of plasma membrane or promoted nucleus swelling. To analyze this, we performed nuclear staining with Hoechst 33342. Hoechst 33342 is able to penetrate the plasma membrane of live cells, while DAPI is not. Intriguingly, TXNIP overexpression did not affect the number of Hoechst 33342‐positive cells (Fig. 3C). These results suggest that nuclear size did not change, and the permeability of cell membrane was enhanced by increased expression of TXNIP.

### Growth inhibition caused by TXNIP overexpression involves autophagy in MLL‐r AML cells

**Fig. 4:**
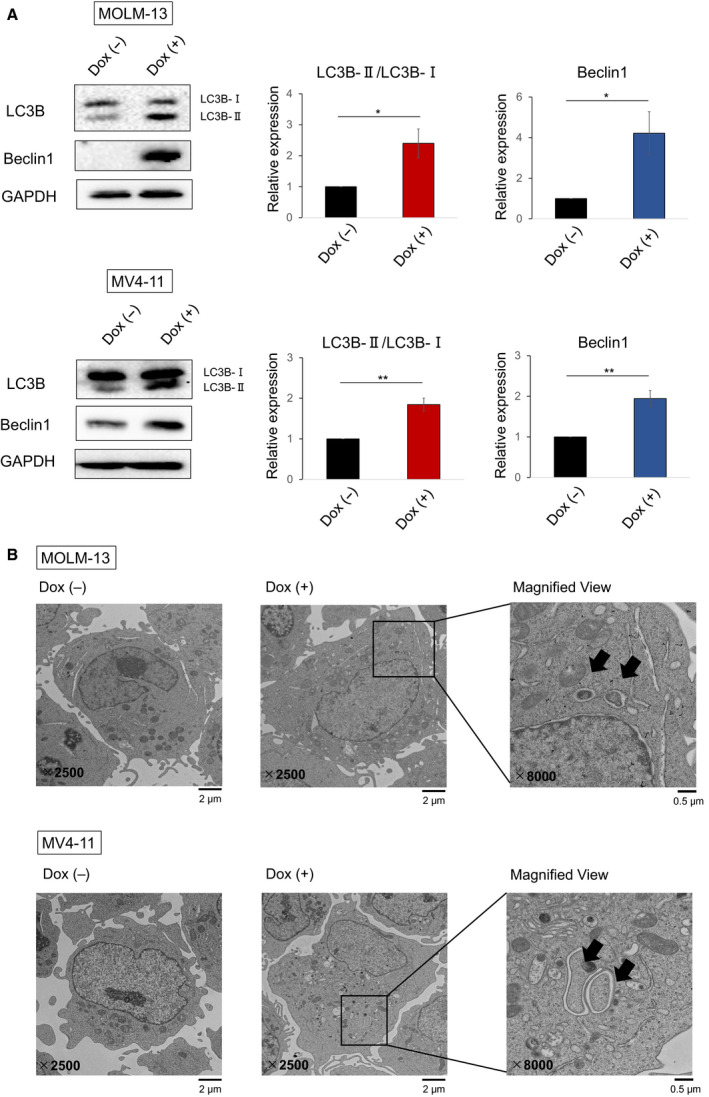
TXNIP induces autophagy in MLL‐r AML cells. (A) Immunoblot analysis showing increased lipidated LC3B (LC3B‐II, bottom band) and Beclin 1 in TXNIP‐overexpressed MOLM‐13 and MV4‐11 cells. Dox‐on‐dependent TXNIP‐overexpressed MOLM‐13 and MV4‐11 cells were treated with or without 3 μm doxycycline. Cell lysates were prepared and analyzed by immunoblotting with the indicated antibodies 72 h after treatment (*n* = 3). GAPDH was used as loading control. (B) TEM analysis of TXNIP‐overexpressed MOLM‐13 and MV4‐11 cells. Dox‐on‐dependent TXNIP‐overexpressed MOLM‐13 and MV4‐11 cells were treated with or without 3 μm doxycycline. Seventy‐two hours after treatment, cells were harvested and examined by TEM. Representative magnified images are shown in the right. The black arrows in the image indicate typical double‐layer membrane autophagosomes. Scale bars, 2 or 0.5 μm. Images are representative images of two reproducible independent results. Data are expressed as mean ± SEM values. *P < 0.05, **P < 0.01, by 2‐tailed Student’s *t‐*test.

**Fig. 5:**
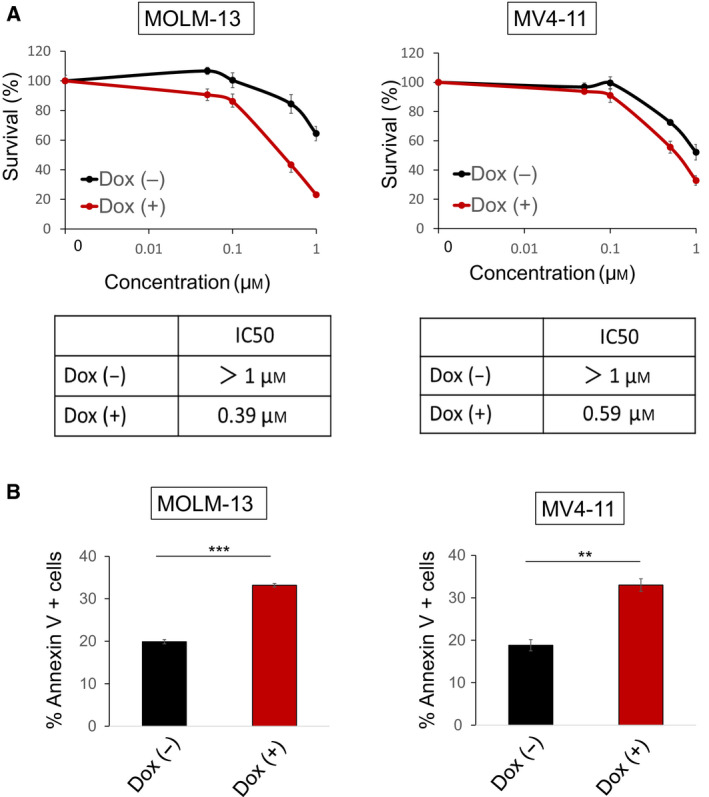
TXNIP enhances the effect of ABT263. (A) IC50 value of ABT263 in dox‐on‐dependent TXNIP‐overexpressed MOLM‐13 and MV4‐11 cells treated with or without doxycycline (*n* = 3). Dox‐on‐dependent TXNIP‐overexpressed MOLM‐13 and MV4‐11 cells were treated simultaneously with 3 μm doxycycline and various concentrations of ABT263. Forty‐eight hours after treatment, IC50 values were calculated. (B) TXNIP enhanced ABT‐263‐induced apoptosis. Dox‐on‐dependent TXNIP‐overexpressed MOLM‐13 and MV4‐11 cells were treated simultaneously with 3 μm doxycycline and 300 nm ABT263. Forty‐eight hours after treatment, the number of cells stained with annexin V was scored by flow cytometric analysis (*n* = 3). Data are expressed as mean ± SEM values. **P < 0.01, ***P < 0.001, by 2‐tailed Student’s *t‐*test.

Since TXNIP is a critical regulator of metabolism and fasting response, we assumed that TXNIP inhibits MLL‐r AML cell growth by inducing autophagy. Immunoblotting analysis revealed that TXNIP overexpression markedly upregulates Beclin 1 and LC3B‐II, both of which are the biomarkers of autophagy (Fig. 4A. A classic autophagy detection tool, TEM, also showed the formation of autophagosome in TXNIP‐overexpressed MOLM‐13 and MV4‐11 (Fig. 4B). These results indicate that TXNIP does not promote apoptosis but autophagy. It has been reported that Beclin 1 inhibits apoptotic signaling in AML cells by enhancing Bcl‐xL binding to Beclin 1 and reducing Bad‐Bcl‐xL complex that promotes apoptosis [33]. Therefore, we tested the effect of ABT263, a BH3 mimetic that targets Bcl‐2 and Bcl‐xL proteins [34], on TXNIP‐overexpressed MOLM‐13 and MV4‐11 cells. Notably, TXNIP overexpression in MLL‐r AML cells enhanced sensitivity to ABT263 (Fig. 5A,B). Therefore, our present study shows that TXNIP causes growth arrest and positively regulates autophagy in MLL‐r AML cell lines, enhancing the effect of ABT263.

## Discussion

Recent studies have revealed that TXNIP is silenced in a wide range of cancer cells. TXNIP is downregulated in bladder cancer based on the grade and stage [35]. Moreover, low TXNIP expression is reported as a potent prognostic biomarker for gastric cancer recurrence [36]. These findings suggest that depletion of TXNIP endows cancer cells with survival and growth advantages. Thus, we assumed that TXNIP expression might be decreased in AML with high‐grade malignancy. Prognostic analysis using clinical datasets revealed that lower TXNIP expression is associated with poor prognosis in refractory AML and relapsed AML. Zhou *et al*. [20] described that TXNIP is epigenetically repressed in AML cells. However, it has been unknown whether the expression of TXNIP varies among the subtypes of AML. We analyzed clinical datasets and examined the relationship between TXNIP expression and common chromosome abnormalities in AML. Against our expectations, TXNIP expression was increased in AML with t (8; 21) and t (15; 17) (Fig. 1B). However, TXNIP was suppressed in AML with inversion 16 (inv (16)) and MLL rearrangements (Fig. 1B). MLL‐r leukemia, in which TXNIP expression is most decreased, is caused by translocation of 11q23 with more than 30 different chromosomal sites resulting in MLL fusion genes. MLL regulates histone methylation and is involved in chromosome remodeling [2]. Changes in histone methylation patterns due to MLL fusion protein may contribute to the reduction of TXNIP in AML cells.

Inhibition of glucose uptake and activation of apoptosis via reactive oxygen species have been reported as the main mechanisms of tumor suppression by TXNIP; however, the unified molecular mechanism has not been clarified. In this study, we performed TXNIP overexpression experiments in two AML cell lines with MLL abnormalities. MOLM‐13 carries t(9;11) and expresses MLL‐AF9, which is the most common MLL rearrangements, generally accompanied by intermediate risk [37, 38, 39]. MV4‐11 carries t(4;11) and expresses MLL‐AF4, which shows poor prognosis [39]. Interestingly, TXNIP suppressed cell proliferation, but exerted no influence on cell cycle or apoptosis (Fig. 3A,B). Based on these results, we considered that the antileukemic effect of TXNIP involves the mechanisms other than cell cycle arrest and apoptosis. annexin V/DAPI staining revealed that TXNIP overexpression promotes plasma membrane permeabilization (Fig. 3C), which might weaken cells and confers disadvantages for their survival. Further, we suspected autophagy because TXNIP is a crucial regulator of metabolism and response to fasting. We found that TXNIP promotes autophagy by increasing Beclin 1 at the protein level. The most common autophagy inducer is rapamycin, an mTOR inhibitor, but it has clinical risks of adverse side effects [40]. Furthermore, it has been reported that it does not induce autophagy in AML cells as in other cancer cells [41], which suggests that autophagy induction in AML cells may involve different mechanisms from solid tumors. Autophagy suppresses and promotes apoptosis depending on the cellular circumstances [42]. We showed that TXNIP overexpression increases sensitivity of leukemia cells to the Bcl‐2 and Bcl‐xL inhibitor, ABT263, by promoting apoptosis induction (Fig. 5A,B). Meanwhile, TXNIP did not augment the effect of Bcl‐2 selective inhibitor, ABT199 (data not shown). Considering these data and a previous study, which described that Beclin 1 suppresses apoptosis by interacting with Bcl‐xL [33], it is possible that inhibition of Bcl‐xL is essential for the induction of cell death in TXNIP‐overexpressed MLL‐r AML cells. Although further studies are required to verify interaction between TXNIP and Bcl‐2 family, our findings indicated that TXNIP overexpression‐induced autophagy increases sensitivity to ABT263.

In conclusion, we demonstrated that TXNIP inhibits leukemogenesis possibly via induction of autophagy, and a combination treatment with Bcl‐xL inhibitors is highly effective in promoting apoptosis. These findings have the potential to establish a promising therapy against refractory AML with MLL rearrangements.

## Conflict of interest

The authors declare no conflict of interest.

## Author contributions

MN designed research, performed experiments, analyzed data, and wrote the manuscript. AK helped to collect data. HM and SA participated in discussions and interpretation of the data and results and commented on research direction. HM initiated the study, supervised research, and gave the final approval for submission.
